# Points of Interest in the Care of Children's Teeth

**Published:** 1897-01

**Authors:** B. J. De Vries

**Affiliations:** Holland, Mich.


ARTICLE V.

### POINTS OF INTEREST IN THE CARE OF CHILDREN'S TEETH.

BY B. J. DE VRIES, D. D. S., HOLLAND, MICH.

I will include under this subject children between the ages of four and fourteen, as that is the period of life that great depredations usually exist in the oral cavity.

The prevailing idea is that when a boy or girl has grown up to manhood or womanhood, then they must have their teeth looked after, as that is the period of life when selfrespect is generally asserted.

Young men and women call upon a dental practitioner sooner and oftener on account of their personal appearance than from a sense of the utility of the organs of mastication. Call to mind your appointment book and note your cases. Here is one who says: "I am going to have my teeth attended to. What can you do with this or that tooth? Is it worth saving? or shall I have a plate? Or, perhaps, mother says: "I must have a gold filling in my front teeth?" She wants nothing else.

There is another class of patients that are driven to our offices with the toothache, and we exert an influence on them that is beneficial for the preservation of these organs, for they heed our warning and have their teeth cared for and saved.

The deplorable condition the dentist is called upon to witness, the putrefaction, disease and suffering, both in young and old, can, to a large extent, be traced back to boyhood or girlhood, before the age of fourteen. Show me a boy or girl at the age of fourteen with all the teeth *in situ*, free from decay or properly filled, correctly articulated and properly cared for, and I will show you a man or woman who will point with pride to his dentist and who will be a blessing to his offspring and a comfort to himself.

Then, since our boys and girls of to-day twenty years from now will be our men and women, filling all vocations of life, it becomes evident that if we desire a race with well-cared for and sound teeth, *childhood* is the time to train, to regulate, to prune, before the destructive agencies or malpositions will stamp upon that face the marks of neglect of bygone days. The gardner deals tenderly with his young sapling as its top towers heavenward—he bends, prunes and cultivates that he may obtain a tree of symmetry and beauty whose shade will be sought by many a weary traveler.

We, as dentists, whose duty it is to care for children's teeth when diseased, to watch with great care the permanent teeth advance and take the places of the temporary teeth, have a higher duty to perform, a duty that may not bring us directly any dollars and cents, a duty that costs us nothing which we can perform as we are about our daily business—the education of our patients in regard to the dental organs.

Every mother ought to know that at the age of six, or thereabouts, the first permanent molar makes its appearance immediately back of the deciduous molar. She must be instructed about the value of this tooth during that critical period of childhood, that the second molar will not appear until the twelfth year, and that the child will have to depend upon this tooth, to a great extent, for mastication.

It is a universal rule that parents will mistake this tooth for a deciduous molar, yes, and even eight out of every ten physicians will make the same mistake.

Note the surprise which is manifested when we tear, with cold steel, an abscessed, ugly-looking molar from its hiding-place in the mouth of an eight year old child who could rest neither day nor night, whose moaning and earache, through reflex neurosis, perplexed both parents alike; and it remained an open question whether the child really had the mumps or whether a tooth might not have been the cause of all this suffering.

The first question after the removal of the cause is, "Doctor, will she get a new one for that?" "Where ignorance is bliss, it is folly to be wise," and if we think it will shock the parents too severely we can safely answer this question in the affirmative, for we know that the twelfth year molar will get there some way, at all hazard, if it has to get there head foremost.

This total ignorance of the public in regerd to the dental organs is deplorable and how best to educate our people in the care and preservation of the teeth is an open question to be solved by the dental profession of to day. A comparatively small part of any community comes in contact with a dentist and have established habits of daily using the brush, and especially is this among our children from four to fourteen years. Where parents do not enforce these habits, and interest themselves concerning the welfare of their children how can we expect a better condition.

It is also true that in spite of all efforts some teeth will not stand the test of time, and we will not deny that a perfect and healthy set of teeth can exist in spite of all neglect. These are exceptions, however, and not the rule. Nature, unassisted, is as mysterious as nature, assisted, is wonderful. A child may pass through the period of eruption of the deciduous teeth without an ache or pain. These organs may perform their functions perfectly and in due time be replaced by permanent teeth as perfect and symmetrical as the former. But, alas! this is not always so. Too often the skill of the dentist is taxed to its utmost in arresting decay and preserving the beauty and symmetry of the face.

How, then, shall we educate the public along this line? The dental profession itself is inadequate to the task; so we must look elsewhere for aid. Where better can we go for assistance than to that grand educator itself—the public schools of our land. Already there has been added to the curriculum of our schools such branches as hygiene and physiology. Why not also give instruction, in a practical way, that can be comprehended by the young in the care of the teeth? If one hour in every week was devoted to this subject, and the subject rightly handled, it would be time well-spent.

Such questions as these taught from charts with few illustrations, would fill a long-felt want.

Give the number and names of the deciduous and permanent teeth. Time of eruption. What kind of food is beneficial to tooth structure?

How should teeth be brushed? With what? Etc., etc.

I believe, if some wide awake dentist would write a small, attractive work on this subject to be used in the public schools, he could confer a great benefit upon society, and I am sure it would not be without remuneration to himself. Society must learn that a dentist can not save teeth without the assistance of their own efforts in caring for the same. There are those who will not, among men and women as well as children, but let us do something for those that know not how and why, who never come near our offices, and who are waiting to be taught that they may care for their own teeth as far as in their power lies, and to be able to discern when they need the assistance of a dentist.

We take it, then, for granted, that if we expect to see good results from our labors we must establish habits of cleanliness. The use of the brush and a proper antiseptic mouth-wash are essential parts to be performed by our young patients. And without these habits our work will be null and void.

On the other hand, the surgical and mechanical part to be performed by the dentist is of great importance.

Because the deciduous teeth are only for temporary service is no reason why they should not be properly cared for and filled. We can expect far better permanent teeth, and better development of the maxilla where the deciduous teeth are well cared for and properly exercised, and it should be the aim of every dentist to give this subject the utmost attention however arduous the task may be.

The approximal surfaces of the deciduous molars are most frequently attacked by decay, and if not filled in time these spaces will form most acceptable places for microbes of fermentation and putrefaction. Of all suffering we are called upon to relieve, in nine cases out of ten, it is in this region, in connection with the first permanent molar.

It is my practice to fill these temporary teeth with cement only, and as soon as possible after decay has attacked them.

I never extract them if I can combat the difficulty in some other way. Very often I have found after much ulceration fungus growths upon the gums, and the apical portion of the roots exposed. In such cases, as also in the incisors, I nip off the exposed roots, leaving the crowns *in situ*, to fill up the space and favor the expansion of the maxilla.

I have heard of dentists devitalizing a deciduous tooth with arsenic and filling the roots with gutta percha. I must confess that I never attempted such an operation on account of the difficulty in managing children, and the many obstacles we have to contend with in manipulating these delicate tissues.

Wherever I find it necessary to save a deciduous tooth, where the nerve is decomposed, I render it aseptic with peroxide and cloves and carbolic acid ½ and ½. After that I fill the roots with shreds of cotton impregnated with aristol dissolved in chloroform and fill the cavity with cement.

My reason for filling these roots with cotton is that it is next to impossible to apply enough hot air so as to get them perfectly dry, and if there should be any septic matter left in the canals, cotton will more readily absorb it, and the aristol will render it aseptic.

What to do with the first molar is another perplexing question. A great many of our young patients have to lose this tooth at an early age. The best time to extract the same we will not here discuss but we will say, that many of them are extracted that should be saved.

This is the most useful and best adapted tooth for mastication of all the dental organs. Upon the loss or retention of this tooth hinges many intricate problems of irregularity and articulation. When the structure of this tooth is fairly good, and if attacked by decay at all, the most favorable surface is invariably the anterior proximal; the reason for this can be readily seen when there is decay in the posterior proximal surface of the second deciduous molar. Food will be impacted between these two teeth, and through firmentation the first molar will be affected at the anterior proximal surface.

The question is often asked, should, in such cases, the tooth be filled with a permanent filling?

We believe that if the tooth structure is good, and there is a chance to make a perfect operation, we should not hesitate to fill this tooth with gold; but the chances are that we find decay at the seventh and eighth year, and the second deciduous molar will not be shed until the twelfth year which makes it difficult of access.

In such cases I fill this tooth with a phosphate filling, and as soon as the second deciduous molar is shed, and before the second permanent bicuspid is erupted, I fill the first permanent molar with a permanent filling. This method obviates the necessity of cutting through the grinding surface, and in many cases can be accomplished without the rubber-dam. For, let us remember that all operations on the teeth of children should be conducted with the utmost of care and as painless as possible.—*Dental Register.*

